# Lung Perfusion Scintigraphy Reveals a Duplicated Superior Vena Cava With a Right-to-Left Shunt: A Rare Vascular Variant

**DOI:** 10.7759/cureus.57702

**Published:** 2024-04-06

**Authors:** Joshua D Zamarripa, Horace Hayes, Michael R Povlow

**Affiliations:** 1 Transitional Year Medicine, San Antonio Uniformed Services Health Education Consortium, San Antonio, USA; 2 Radiology, San Antonio Uniformed Services Health Education Consortium, San Antonio, USA; 3 Radiology, General Leonard Wood Army Community Hospital, Fort Leonard Wood, USA

**Keywords:** nuclear medicine imaging, rare anatomical variants, right-to-left shunting, lung perfusion scintigraphy, duplicated superior vena cava

## Abstract

Lung perfusion scintigraphy is a common nuclear medicine exam performed for the evaluation of pulmonary emboli, often in the emergency setting. There can be confusion when a radiotracer is located outside of the normal physiologic distribution. This can occur due to improper radionuclide tagging or may be due to anatomic variations. We present a case where a patient presented with bilateral lower extremity deep vein thrombosis and a nuclear medicine lung perfusion scintigraphy showing a complete right-to-left shunt related to a rare anatomical variant of a duplicated superior vena cava (SVC) with the right SVC draining directly into the systemic circulation via the left atrium.

## Introduction

The most common indication for lung perfusion scintigraphy is for the evaluation of pulmonary emboli [1]. During such a perfusion scan, technetium radionuclide tagged to macroaggregated albumin (99mTc-MAA) particles are injected peripherally and migrate via the right heart circulation to the lung capillaries. Because of the size of the particles (5-100 microns), they occlude the pulmonary capillary bed, which then allows for the gamma-emitting technetium radionuclide to be imaged using a gamma camera. In a patient without a pulmonary embolism (PE), there should be diffuse radiotracer uptake throughout the lungs, without any photopenic defects. In a patient presenting with a pulmonary embolism, the particles are unable to travel beyond the occlusive thrombus, leading to segmental photopenic defects in the lung parenchyma due to the inability for tracer to get to the capillary beds [2]. After injection, the only region that should show radiotracer uptake is the lungs, as normal circulation brings the tracer from the venous system into the right atrium, through the right ventricle, and into the pulmonary artery, where the particles then travel downstream before becoming lodged within the pulmonary capillary beds. Therefore, no systemic uptake should be seen, except in the case of free 99mTc not bound to MAA or if a right-to-left shunt is present [1,2].

We describe a case of an elderly female presenting for suspected pulmonary embolism who was found to have a rare vascular abnormality leading to a complete right-to-left shunt, which was identified with lung perfusion scintigraphy.

## Case presentation

**Figure 1:**
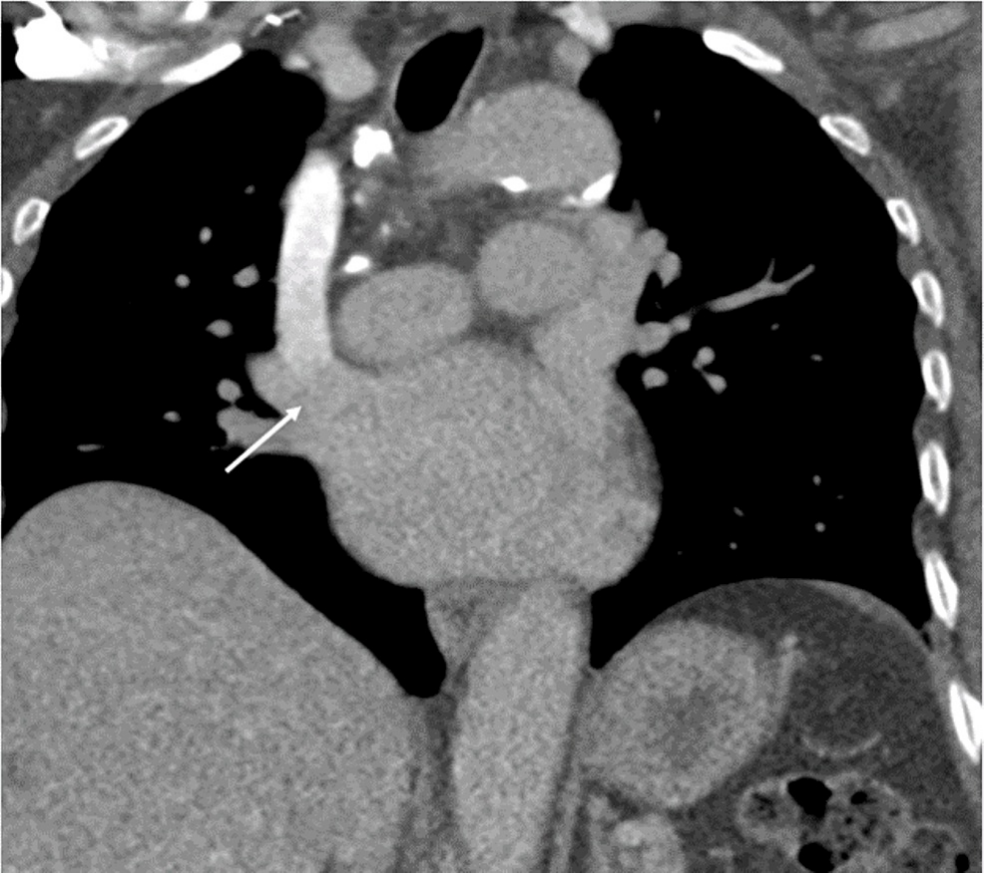
Coronal maximum intensity projection (MIP) image of the chest from the initial CT angiogram injected through the right upper extremity. There is no contrast within the vasculature of either lung. The contrast bolus is seen entering the heart directly from what is assumed to be a normal right-side SVC (white arrow). SVC: superior vena cava.

A 75-year-old female with a history of pulmonary embolism and two previous cerebrovascular accidents presented to the Brooke Army Medical Center emergency department on Ft. Sam Houston in San Antonio, TX, by personal vehicle seeking evaluation for bilateral calf tenderness, fatigue, and dizziness at rest following a recent eight-hour car ride. An elevated d-dimer in the emergency department prompted a duplex ultrasound of bilateral lower extremities, which revealed deep vein thrombosis (DVT) in the left mid-femoral vein and the left popliteal vein. The patient was subsequently admitted by the internal medicine (IM) team for anticoagulation. Given her prior history and the presence of DVTs, a work-up for pulmonary embolism (PE) was started. The initial computed tomography (CT) pulmonary angiogram with injection of IV contrast into a peripheral IV in the right upper extremity was nondiagnostic for PE as, interestingly, there was no contrast seen in the vasculature of either lung even though a contrast bolus was seen in the superior vena cava (SVC) entering the heart (Figure 1).

**Figure 2:**
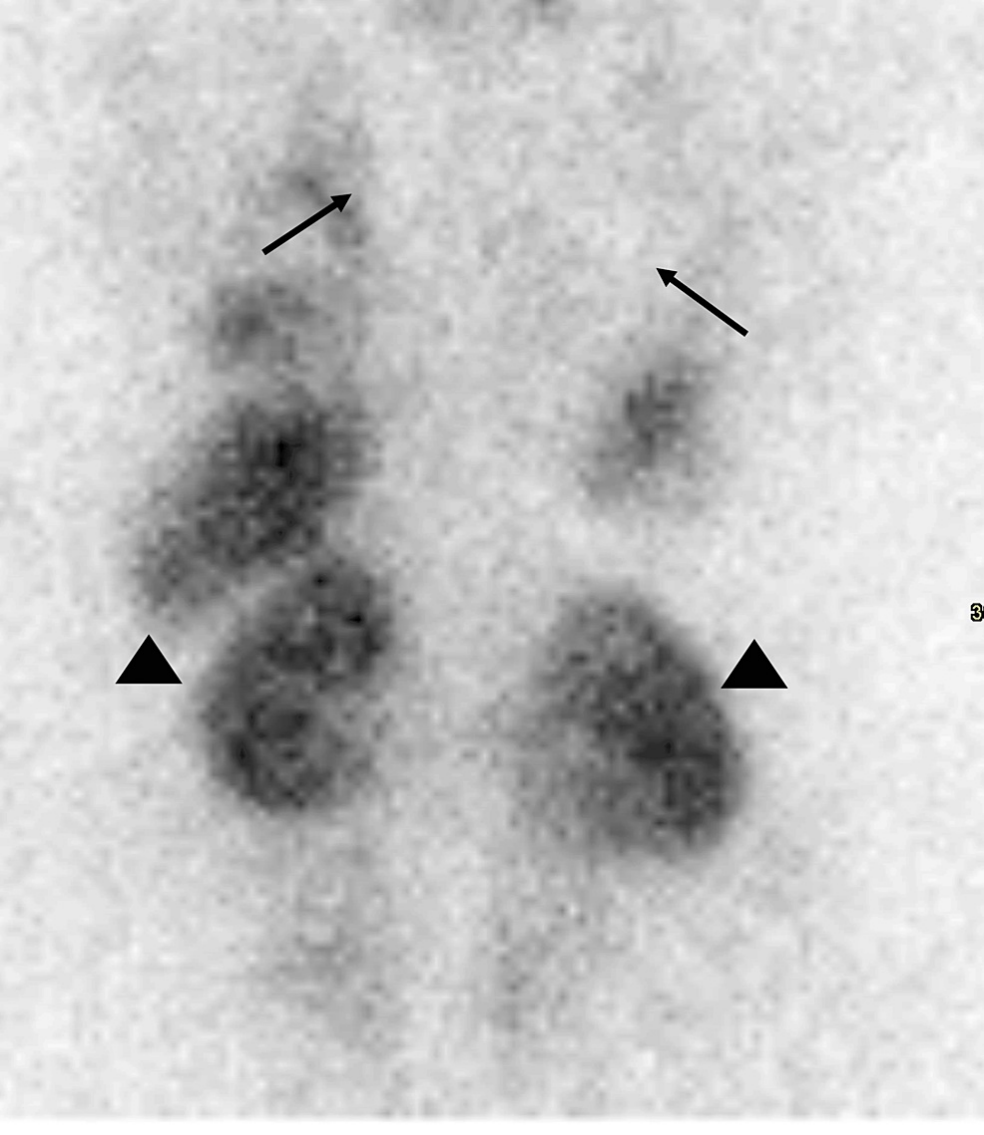
Posterior planar images of the torso after injection of 5.5 mCi 99mTc-MAA. There is no radiotracer uptake within the lungs (black arrows). Radiotracer uptake is noted within the kidneys (black arrowheads), spleen, liver, and myocardium. Patient weight: 208 lbs. BMI: 32.6 kg/m^2^.

**Figure 3:**
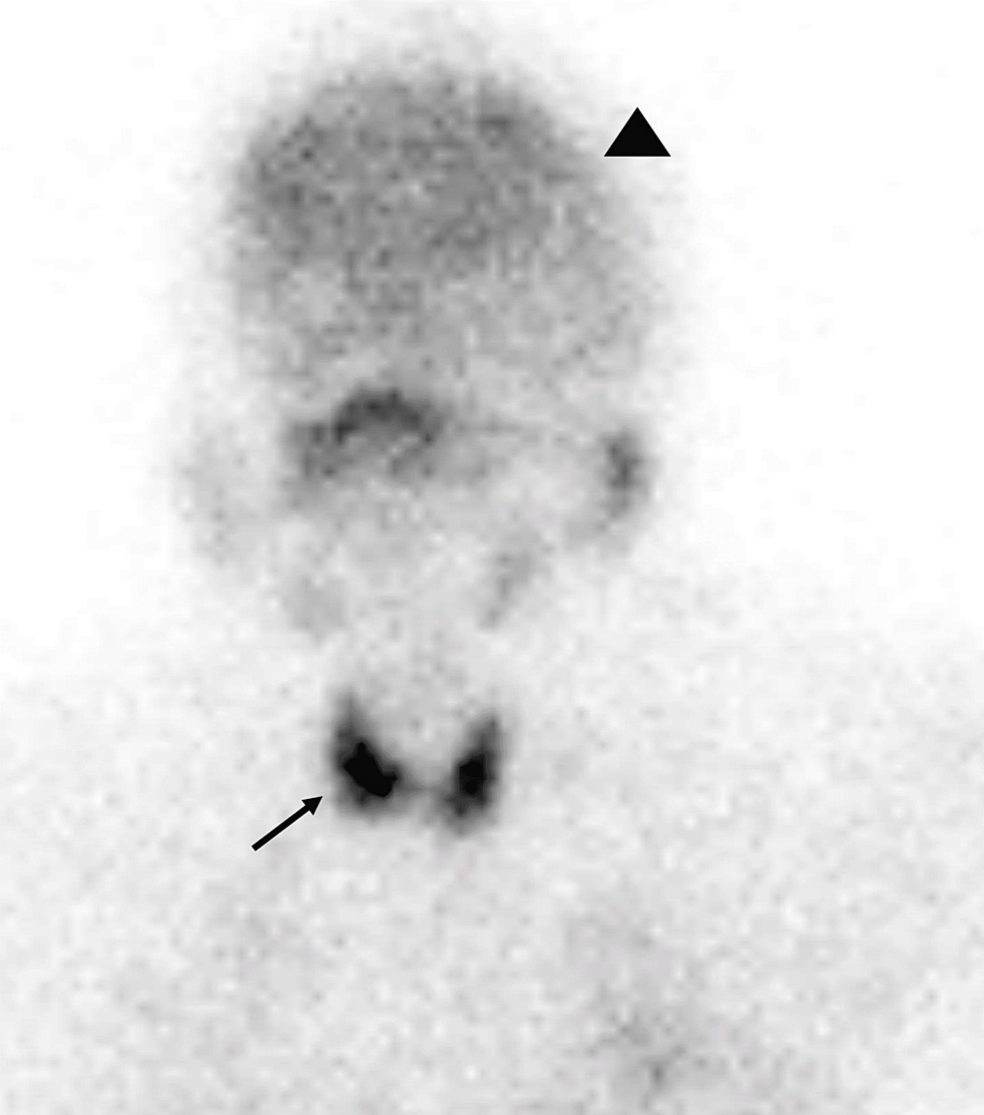
Anterior planar images of the head and neck after injection of 5.5 mCi 99mTc-MAA. There is radiotracer uptake within the thyroid (black arrow), brain (black arrowhead), and salivary glands. Patient weight: 208 lbs. BMI: 32.6 kg/m^2^.

**Figure 4:**
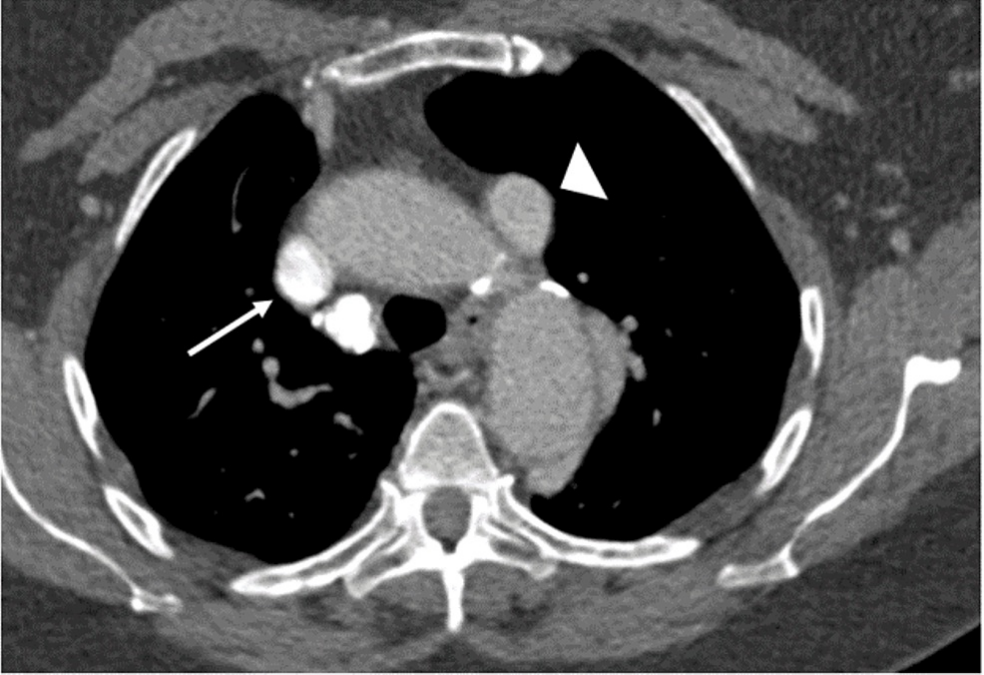
Axial CT image from the initial CT angiogram of the chest injected through the right upper extremity. The right (white arrow) and left (white arrowhead) SVC are both seen on this slice, with the more dense contrast bolus seen in the right SVC. SVC: superior vena cava.

Given the degraded nature of this study to evaluate for PE, perfusion-only lung scintigraphy was ordered by the IM team and performed by BAMC nuclear medicine. The 99mTc-MAA was injected into the same peripheral IV in the right upper extremity. The scan demonstrated radiotracer uptake within the myocardium, thyroid, stomach, and kidneys. No radiotracer was seen within the lungs (Figure 2). Given an atypical distribution of radiotracer with uptake in systemic circulation organs, scintigraphic brain imaging was performed to assess for cerebral uptake, which was confirmed (Figure 3). The results of these nuclear medicine studies were consistent with a right-to-left shunt, which prompted a review of the initial CT pulmonary angiogram to look for a missed cause of this shunting. Though previously missed, the right-sided SVC was now appreciated to be draining directly into the left atrium rather than the normal drainage pathway of the right atrium, accounting for the right-to-left shunt (Figure 1). A previously missed duplicate left-sided SVC was then noted on this initial study as well (Figure 4).

**Figure 5:**
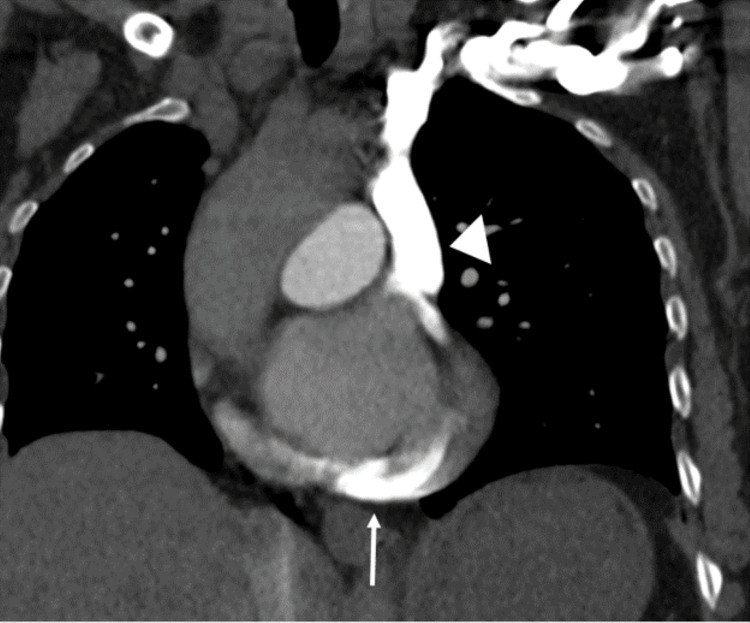
Coronal MIP image of the chest from the second CT angiogram injected through the left upper extremity The contrast bolus is seen entering the right atrium via the left-sided SVC (white arrowhead) through the coronary sinus (white arrow). MIP: maximum intensity projection; SVC: superior vena cava.

A follow-up CT pulmonary angiogram was then performed, given persistent concern for a pulmonary embolism, with contrast injection through a new intravenous catheter in the left upper extremity. This showed the contrast bolus going into the left-sided SVC, through the coronary sinus, and draining into the right atrium (Figure 5). The bolus timing was appropriate for the evaluation of a pulmonary embolism, which was absent in this patient. A follow-up cardiac MRI again demonstrated these anatomic findings and demonstrated a right-to-left shunt and an echocardiogram with agitated saline (bubble study) showed no intracardiac shunt lesion.

## Discussion

We report a rare case of a complete right-to-left shunt of the superior circulatory system in a 75-year-old female due to a rare variant of duplicated SVC through lung perfusion scintigraphy, which was subsequently confirmed with CT pulmonary angiography.

As stated before, no systemic uptake of 99mTc MAA should be seen on lung perfusion scintigraphy except in the case of free 99mTc (not bound to MAA) or a right-to-left shunt. Thus, if a radiotracer is identified in areas other than the lungs, as seen in this case, it is imperative to further assess whether poor labeling of the MAA occurred or, less commonly, whether a right-to-left shunt is present. When poor labeling occurs, free 99mTc (not bound to MAA) will not follow the normal MAA distribution pattern and can be seen in areas such as the thyroid, stomach, salivary glands, or kidneys. Free 99mTc, however, will not deposit in the brain, so if there is uptake in these other tissues, scintigraphic imaging of the brain should be performed to confirm a right-to-left shunt. In this case, as seen in Figure 3, this patient clearly has the uptake of 99mTc in the myocardium, thyroid, stomach, and kidneys. Additionally, however, as seen in Figure 4, there is also an uptake of radiotracer seen in this patient's brain. This confirms that the systemic uptake of the radiotracer in this patient does indeed represent a true right-to-left shunt and is not a result of free 99mTc [1,2].

If a right-to-left shunt is confirmed with lung perfusion scintigraphy, evaluation for the etiology of the shunt should be performed by either reviewing prior cross-sectional imaging or acquisition of additional imaging. The causes for right-to-left shunt can be intracardiac (e.g., an atrial or ventricular septal defect) or extracardiac (e.g., patent ductus arteriosus or another anomaly of the thoracic vasculature). These can be assessed using one or a combination of computed tomography (CT), magnetic resonance imaging (MRI), or echocardiography [2].

As confirmed by the two CT pulmonary angiograms obtained throughout this hospitalization, the right SVC of this patient carried the bolus of radiotracer from the right upper extremity IV directly into the left atrium without communication to the right atrium, resulting in the entirety of the 99mTc-MAA particles bypassing the lungs. This resulted in the impressive and somewhat confusing imaging presented, which is consistent with complete right-to-left shunting. If the initial bolus of the radiotracer was injected into the left upper extremity instead, the 99mTc-MAA particle would have been carried from the left upper extremity to the right atrium via the coronary sinus and into the lungs. If this had been the case, the initial CT pulmonary angiogram would have been sufficient to rule out PE and the subsequent investigation with lung perfusion scintigraphy would likely not have been prompted, resulting in a missed diagnosis of SVC duplication [1,2]. This may have been why this patient had not been diagnosed with duplicate SVC previously.

SVC duplication occurs in 0.3% of the general population [3]. This abnormality arises due to variations in the development of the embryonic thoracic venous system, resulting in a persistent left-sided SVC in addition to the physiologic right-sided SVC [3]. Various configurations of duplicate SVC have been reported, but most commonly, the right-sided SVC directly enters the right atrium and the duplicate left SVC enters the right atrium via the coronary sinus [3]. Very rarely, as in our case, the right SVC will drain directly into the left atrium, partially or completely, and result in a right-to-left shunt [4]. When these types of anomalies present at a relatively older age, the malformation is well tolerated; however, they are predisposed to systemic emboli (as in this patient with prior CVAs) and the development of brain abscesses [5]. To our knowledge, this is the oldest reported age of presentation [6].

## Conclusions

As the oldest known presentation of this anatomical variant, this adds to the literature an instance of duplicate SVC diagnosis beyond childhood using nuclear medicine techniques. The practicing radiologist should be familiar with the diagnosis of pulmonary emboli on perfusion imaging and also be able to recognize abnormal physiologic uptake and differentiate between poor 99mTc labeling and a right-to-left shunt. The key identifier of radiotracer uptake in the brain led to the correct diagnosis of a right-to-left shunt, which was then confirmed with cross-sectional imaging. If a practicing radiologist is familiar with SVC duplication and includes it in their search pattern, this diagnosis could have been made based on earlier imaging in this and other patients. This diagnosis likely explains her previous strokes, will likely impact further management of this patient, and is thus a should-not-miss (yet easy-to-miss) diagnosis.
